# Comparison of Lidocaine and Dexmedetomidine in Preventing Fentanyl-induced Cough in Patients Undergoing Elective Surgery: A Blinded Randomized Controlled Trial

**DOI:** 10.5812/aapm-167186

**Published:** 2026-01-04

**Authors:** Hashem Jarineshin, Hananeh Zarezadeh, Sara Ghazizadeh, Masoomeh Mahmoudi, Fatemeh Taherzadeh, Mobina Vatankhah

**Affiliations:** 1 Department of Anesthesiology, Critical Care and Pain Management Research Center, Hormozgan University of Medical Sciences, Bandar Abbas, Iran; 2 Clinical Research Development Center of Shahid Mohammadi Hospital, Hormozgan University of Medical Sciences, Bandar Abbas, Iran; 3 Student Research Committee, Faculty of Medicine, Hormozgan University of Medical Sciences, Bandar Abbas, Iran; 4 Cardiovascular Research Center, Hormozgan University of Medical Sciences, Bandar Abbas, Iran; 5 Department of Research and Technology, Hormozgan University of Medical Sciences, Bandar Abbas, Iran

**Keywords:** Dexmedetomidine, Lidocaine, Fentanyl-Induced Cough

## Abstract

**Background:**

Fentanyl-induced cough (FIC) refers to a cough that can occur as a side effect of fentanyl, an opioid analgesic primarily used for pain management. Both lidocaine (LIDO) and dexmedetomidine (DEX) are effective in preventing FIC, but the comparative efficacy of each has not yet been thoroughly investigated.

**Objectives:**

In this study, we aimed to compare the effect of LIDO versus DEX in preventing FIC in adult patients undergoing elective surgery.

**Methods:**

A total of 171 adult patients with American Society of Anesthesiologists (ASA) class I or II undergoing elective surgery were enrolled in this blinded, randomized, placebo-controlled trial. Patients received either 0.5 μg × kg^-1^ of DEX in 10 mL of isotonic saline, 1.5 mg × kg^-1^ LIDO, or a matching placebo (equal volume of 0.9% saline) prior to fentanyl injection. The incidence and severity of cough were recorded within the two minutes following fentanyl administration. Hemodynamic parameters were measured at different time intervals. Statistical analysis was performed using SPSS version 18. The incidence of cough among groups was compared using the Pearson chi-square test, while continuous hemodynamic variables were analyzed using one-way ANOVA followed by Tukey’s post-hoc test. A two-tailed P-value < 0.05 was considered statistically significant. The study was registered at the Iranian Registry of Clinical Trials (IRCT20211023052848N1).

**Results:**

The incidence of cough was 7.0%, 10.5%, and 33.3% in the DEX, LIDO, and saline groups, respectively (P < 0.001). There was no significant difference between the DEX and LIDO groups (P = 0.453), but both were significantly more effective than the control group (P < 0.001 for DEX vs. saline; P = 0.002 for LIDO vs. saline; P < 0.001 for DEX reduced heart rate, systolic and diastolic blood pressure).

**Conclusions:**

The results of this study demonstrated that 0.5 μg × kg^-1^ of DEX and 1.5 mg × kg^-1^ of LIDO are equally effective in preventing FIC. However, hemodynamic parameters are more markedly suppressed in the DEX group compared to the lidocaine group.

## 1. Background

General anesthesia is widely induced using opioids, including fentanyl and its derivatives (1, 2). In addition, fentanyl is administered in procedures such as laryngoscopy and tracheal intubation because of its short onset of action (3). Intravenous (IV) administration of this drug and its analogs can sometimes be accompanied by a cough that occurs within 2 minutes after injection, known as fentanyl-induced cough (FIC) (4). The prevalence of FIC among patients after general anesthesia is 82.5% (5, 6). The FIC can result in increased intra-abdominal, intracerebral, and intraocular pressure, which may worsen the risk of ocular penetrating injuries and cerebral aneurysm rupture (4). Dexmedetomidine (DEX) and lidocaine (LIDO) have shown potential efficacy in mitigating FIC (7, 8). The DEX is a potent drug with sedative, analgesic, and anti-opioid effects. It causes minimal respiratory depression and can reduce sympathetic outflow and stress (9, 10).

In a study by He et al., the use of DEX reduced the incidence of FIC, but it was not effective in decreasing cough severity. However, the studies conducted so far do not provide sufficient evidence to prove the efficacy of DEX in preventing FIC (8). The LIDO is one of the oldest drugs used in anesthesia. In addition to its well-known local anesthetic and antiarrhythmic properties, IV LIDO has shown numerous beneficial effects in the perioperative period, including anti-inflammatory, analgesic, opioid consumption reduction, and neuroprotection, as well as reduction of postoperative cognitive impairment (POCD) (11). The LIDO has been widely studied as an agent to prevent FIC. Pre-administration of IV LIDO prior to induction has been shown to attenuate airway reflexes and reduce fentanyl-related adverse effects, including cough (12).

To reduce airway reflexes during endotracheal intubation, extubation, bronchoscopy, and laryngoscopy, IV LIDO has been utilized extensively. Prior research has demonstrated that IV LIDO can considerably lessen coughing in sedated patients that is brought on by airway manipulation and stimulation. Administering LIDO (1.5 mg/kg) one minute prior to fentanyl decreased the incidence of FIC from roughly 34% to 13% in a placebo-controlled trial, indicating a significant decrease in cough reflex sensitivity. These results confirm that LIDO is a useful and feasible treatment for coughs brought on by fentanyl (13). However, despite extensive research, the optimal use of LIDO remains a subject of ongoing debate. Moreover, LIDO is not officially approved as a treatment for FIC (7).

## 2. Objectives

We aimed to conduct a blinded, randomized, placebo-controlled trial to compare the efficacy of LIDO, DEX, and a control group in preventing FIC among patients undergoing elective surgery with general anesthesia.

## 3. Methods

### 3.1. Study Design and Participants

This study was a single-center, blinded, randomized clinical trial conducted on 171 patients undergoing elective surgery of any type at Shahid Mohammadi Hospital in Bandar Abbas, Iran. The study was registered at the Iranian Registry of Clinical Trials (IRCT20211023052848N1) on November 17, 2021. Eligible participants were recruited from patients scheduled for elective surgery who met the following inclusion criteria: American Society of Anesthesiologists (ASA) class I or II, aged 18 to 65 years, and provided informed consent. Patients undergoing emergency surgery, those with a history of allergy to fentanyl, DEX, or LIDO, those with a history of asthma or respiratory diseases in the last 4 weeks, and patients receiving corticosteroids were excluded from the study. Participants were randomly assigned to three parallel groups using Random Allocation Software version 1.0.0: The DEX, LIDO, and placebo group (SALINE). Study medications were prepared by a certified anesthesia technician. Assessment and evaluation of cough were performed by an anesthesia resident. Both individuals were blinded to group allocation, as well as to the type and method of drug administration, to ensure adequate blinding and minimize assessment bias.

Upon arrival at the operating room, patients were cannulated with an IV cannula. Monitoring of each patient was conducted using electrocardiogram, non-invasive blood pressure, pulse oximetry, and end-tidal carbon dioxide measurement. In the DEX group, ten minutes prior to anesthesia induction, a blinded nurse initiated an IV infusion of DEX at a dose of 0.5 μg × kg^-1^, diluted in 10 mL of isotonic saline (Precedex^™^ 200 mcg/2 mL, Hospira, Inc., Lake Forest, IL 60045, USA). In the LIDO group, 1.5 mg × kg^-1^ of 2% LIDO, diluted in 10 mL of isotonic saline (100 mg/5 mL, Iranhormone Co., Karaj, Iran), was administered intravenously 90 seconds before fentanyl injection. In the SALINE group, an equal volume of normal saline was administered at the same time, similar to the LIDO group. After administration of the study drugs, 2 μg × kg^-1^ of fentanyl (50 μg/mL, Caspian Daru Company, Rasht, Iran) was injected intravenously within 5 seconds for all patients. During the two minutes following the injection, patients were assessed for the incidence and severity of cough. Patients’ hemodynamic parameters, including mean arterial blood pressure (MAP) and heart rate, were measured and recorded at baseline and at 3, 5, and 10 minutes after fentanyl injection. The outcome assessor was masked to the study group allocation. Data collection was conducted using a pre-designed checklist developed by the senior researcher.

### 3.2. Outcome

The primary outcome was the incidence of cough within 2 minutes after fentanyl injection, which was recorded and compared among the three groups. The secondary outcomes included the severity of cough and hemodynamic variables during surgery.

### 3.3. Blinding

To ensure objectivity, all individuals involved in the study, including caregivers, patients, the outcome assessor, and the data analyzer, were blinded to the study group allocation, except for the primary investigator.

### 3.4. Statistical Analysis

SPSS software version 18 was used for statistical analysis. The sample size was calculated based on the primary outcome (incidence of cough). According to El Baissari et al. (14), the incidence of FIC in the control group was estimated at 40%, with an expected 50% reduction in the intervention groups. Assuming α = 0.05, power = 80%, and a 1:1:1 allocation ratio among the three groups, a minimum of 57 participants per group was required. To compare the incidence of cough among groups, the Pearson chi-square test was used. For secondary outcomes involving continuous quantitative variables with a normal distribution, a *t*-test was performed to compare the groups. For variables without a normal distribution, the Mann-Whitney U test was applied. Chi-square or Fisher’s exact test was used to compare qualitative variables. A P-value < 0.05 (two-tailed) was considered statistically significant in all analyses.

### 3.5. Ethics

Each participant was assigned a unique project code during the study. All analyses were performed using these codes, and the initial identifying information was kept secure by the primary investigator. Patients could withdraw from the study at any stage.

## 4. Results

### 4.1. Patient Characteristics

**Figure 1.**
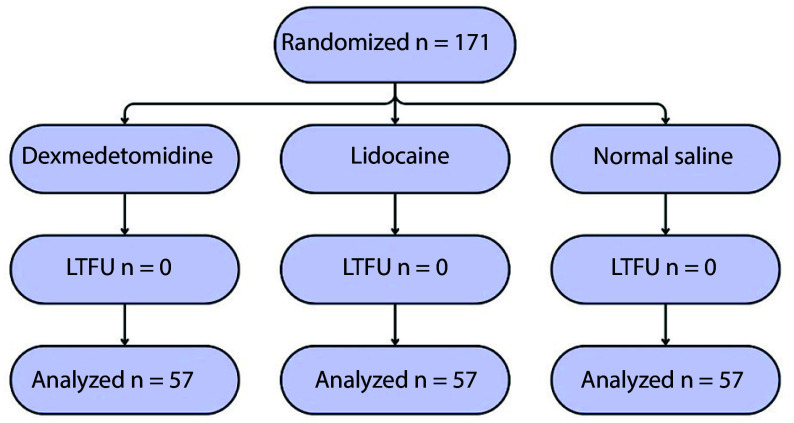
CONSORT flow diagram for the study (abbreviation: LTFU, lost to follow up)

Of the 171 patients studied, 149 (87.1%) were male. The mean age was 32.79 years. There was no loss to follow-up, and all enrolled patients were randomized (DEX group: 57, LIDO group: 57, SALINE group: 57), completed the study, and their data were analyzed (Figure 1). Patients in the groups were comparable in terms of baseline characteristics, and there were no significant differences between the groups. The ASA I included participants without underlying disease (150 patients, 87.7%), and ASA II included participants with mild underlying disease (21 patients, 12.3%; Table 1). 

**Table 1.** Demographic Findings in Three Different Study Groups ^a^

Variables	LIDO	DEX	Saline	P-Value
**Sex **				0.271
Male	48 (84.21)	53 (92.92)	48 (84.21)	
Female	9 (15.79)	4 (7.08)	9 (15.79)	
**Age**	32.94 ± 11.04	33.28 ± 10.22	33.15 ± 12.86	0.953
**ASA**				0.614
I	48 (28.1)	51 (29.8)	51 (29.8)	
II	9 (5.3)	6 (3.5)	6 (3.5)	
**Height (cm)**	170.63 ± 6.99	169.19 ± 6.92	170.105 ± 7.95	0.504
**Weight (kg)**	66.38 ± 11.18	66.24 ± 11.08	66.24 ± 12.93	0.921

Abbreviations: LIDO, lidocaine; DEX, dexmedetomidine; ASA, American Society of Anesthesiologists.

^a^ Values are expressed as mean ± standard deviation (SD) or No. (%).

### 4.2. Clinical Outcomes

The FIC occurred in 12 of the 171 patients (7%). Nine patients (5.3%) had a mild cough (one or two coughs after injection), two patients (1.2%) had a moderate cough (three to five coughs after injection), and only one patient (0.6%) had a severe cough (more than five coughs after injection). The incidence of cough was compared between the groups. Cough incidence in the SALINE group (n = 8) was significantly higher than in the LIDO (n = 2, P = 0.047) and DEX (n = 2, P = 0.047) groups. However, no significant difference was observed between the LIDO and DEX groups (P = 0.691, Table 2). There was no significant difference in the severity of cough among the studied groups (P = 0.736, Table 3). 

**Table 2.** Comparison of Cough Incidence in Different Study Groups Within 2 Minutes After Injection ^a^

Cough Incidences	LIDO	DEX	Saline	P-Value
**Positive**	2 (3.5)	2 (3.5)	8 (14.03)	
L-S				0.047
**Negative**	55 (96.5)	55 (96.5)	49 (85.96)	
L-D				0.691
D-S				0.047

Abbreviations: LIDO, lidocaine; DEX, dexmedetomidine; L-S, lidocaine-saline; L-D, lidocaine-dexmedetomidine; D-S, dexmedetomidine-saline.

^a^ Values are expressed as No (%).

**Table 3.** Cough Severity Between Three Groups ^a, b^

Cough Incidences	LIDO	DEX	Saline	P-Value
**Mild**	2 (100)	2 (100)	5 (62.5)	0.736
**Moderate**	0	0	2 (25)	
**Severe**	0	0	1 (12.5)	

Abbreviations: LIDO, lidocaine; DEX, dexmedetomidine.

^a^ Values are expressed as No (%).

^b^ Mild cough was defined as one or two coughs after injection, moderate cough as three to five coughs after injection, and severe cough as more than five coughs after injection.

**Figure 2.**
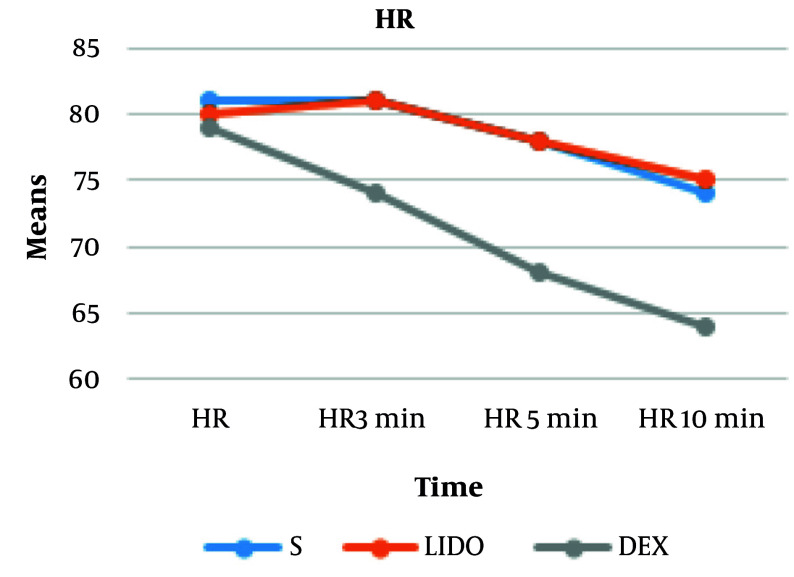
Mean changes in heart rate (HR, beats/min) of patients at different time points [abbreviations: S, saline; DEX, dexmedetomidine; LIDO, lidocaine; values are expressed as mean ± standard deviation (SD).]

**Figure 3.**
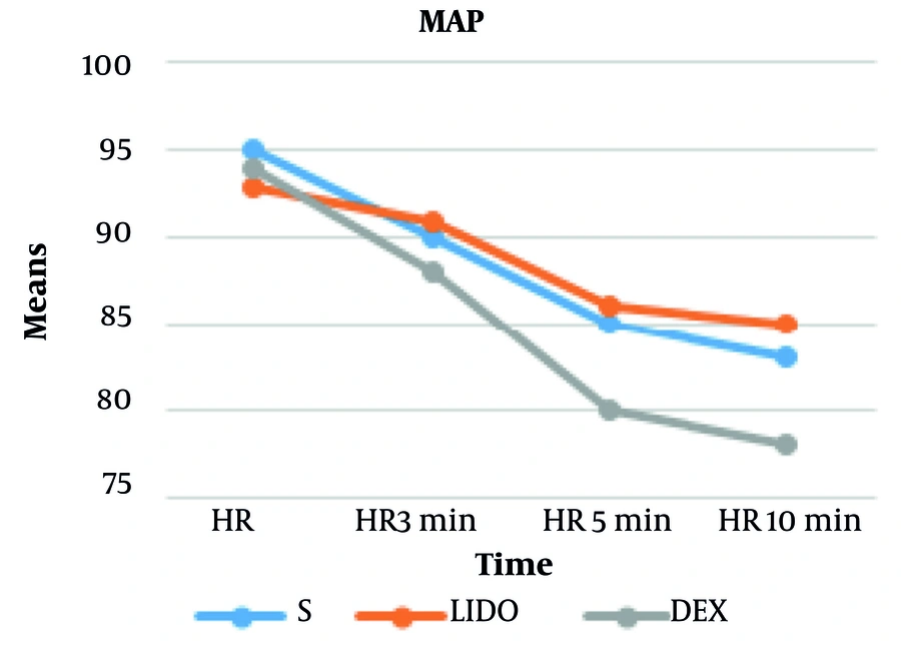
Mean arterial pressure (mmHg) of patients at different time points [abbreviations: MAP, mean arterial blood pressure; S, saline; DEX, dexmedetomidine; LIDO, lidocaine; values are expressed as mean ± standard deviation (SD).]

We observed a significantly lower heart rate at 3, 5, and 10 minutes, and MAP at 5 and 10 minutes, in the DEX group compared to the other two groups. At 10 minutes post-administration, the mean reduction in MAP was 17% in the DEX group, 8.6% in the LIDO group, and 12% in the SALINE group. The mean reduction in heart rate was 19% in the DEX group, 6.2% in the LIDO group, and 8.6% in the SALINE group. The hemodynamic findings of the participants are shown in Figures 2 and 3. Changes in heart rate and mean arterial pressure from baseline are summarized in Table 4 to further clarify hemodynamic trends following fentanyl administration.

**Table 4.** Changes in Heart Rate and Mean Arterial Pressure from Baseline at Predefined Time Points ^a^

Groups and Parameters	Saline	DEX	LIDO	P-Value
**HR (bpm)**				
Baseline	81.68 ± 15.73	79.05 ± 14.81	80.80 ± 15.10	0.578
3 min	81.49 ± 15.47	74.35 ± 13.63	81.21 ± 14.64	0.010 ^b^
N-D				0.027 ^b^
N-L				0.994
D-L				0.035 ^b^
5 min	78.68 ± 11.72	68.24 ± 10.66	78.68 ± 11.23	0.001 ^b^
N-D				0.000 ^b^
N-L				1.000
D-L				0.000 ^b^
10 min	74.71 ± 12.61	64.26 ± 9.05	75.12 ± 11.14	0.001 ^b^
N-D				0.000 ^b^
N-L				0.979
D-L				0.000 ^b^
**MAP (mmHg)**				
Baseline	95.28 ± 14.72	94.12 ± 13.38	93.56 ± 15.34	0.813
3 min	90.73 ± 13.58	88.31 ± 12.45	91.29 ± 12.66	0.420
5 min	85.31 ± 12.84	80.82 ± 10.04	86.50 ± 10.80	0.023 ^b^
N-D				0.088
N-L				0.839
D-L				0.022 ^b^
10 min	83.03 ± 15.36	78.96 ± 10.47	85.35 ± 10.91	0.004 ^b^
N-D				0.192
N-L				0.582
D-L				0.035 ^b^

Abbreviations: DEX, dexmedetomidine; LIDO, lidocaine; MAP, mean arterial blood pressure.

^a^ Values are expressed as mean ± SD.

^b^ Statistically significant.

## 5. Discussion

This randomized controlled trial demonstrated the effect of DEX and LIDO in preventing FIC. The study was conducted at Shahid Mohammadi Hospital in 2021. Twelve patients (7%) experienced FIC, which was lower than the estimated rate reported in previous studies (28 - 65%) (12, 15). The fentanyl dosage used in this investigation was chosen based on the premedication dose that is typically used and advised. To lessen airway irritation, the dosages of LIDO and DEX were also established based on the average and safe levels reported in earlier research. This may help to explain the lower incidence of FIC seen in our investigation (16). Guler et al. reported that the incidence of FIC was 23%. The possible reason for this difference may be the limited number of female participants (17).

The mechanism involved in this undesirable side effect of fentanyl is unclear, but several pathways have been proposed. By stimulating the µ-opioid receptor, fentanyl and its analogs can send signals to the brainstem via either C-fiber vagal receptors in the mucosa of the upper bronchi or fast-adapting airway receptors. Coughing and bronchial constriction may result from these signals activating motor fibers in the vagus nerve. It has been demonstrated that fentanyl increases cough reactions brought on by citric acid in animal models; this effect can be avoided by inhibiting fast-adapting receptors. This suggested mechanism is further supported by the fact that bronchodilators like terbutaline or salbutamol might lessen coughing and bronchoconstriction prior to fentanyl administration (18, 19).

In our study, DEX and LIDO effectively suppressed FIC. The incidence of cough in the normal saline group was 14.03% (8 patients), which was significantly higher than in the other two groups. These findings are consistent with previous research (20-22). Similar results have been reported by Nazemroaya et al., who demonstrated that DEX and LIDO similarly reduced sympathetic responses without causing clinically significant hemodynamic instability. The absence of significant differences in heart rate and blood pressure between the two drugs in their study is consistent with our findings and further supports the hemodynamic safety of both agents during anesthetic induction (23). However, our results contrast with those reported in previous studies (24, 25). This discrepancy may be attributed to variations in patient demographics, such as age and gender, which could influence cough susceptibility and response to premedication. In the study conducted by Saleh et al., it was reported that DEX in combination with ketamine was more effective in preventing FIC than DEX alone (25).

Regarding the severity of cough in the LIDO and DEX groups, two patients from each group had a mild cough. In the control group, eight patients experienced a cough; five of whom had a mild cough, two had a moderate cough, and one had a severe cough. These findings are consistent with the results of previous studies. Ultimately, these two drugs had the same effect in preventing the occurrence of and reducing the severity of FIC (5, 16, 26).

Our findings demonstrated that DEX significantly reduces heart rate and mean arterial pressure after administration compared to the other groups. These results are consistent with those of previous studies regarding hemodynamic parameters (5, 27). However, it was reported that the use of DEX did not cause hemodynamic instability (8, 9, 28).

While DEX is generally considered safe in many studies, it is important to note its potential impact on hemodynamics. The DEX can suppress the release of norepinephrine (10). This leads to reductions in blood pressure and heart rate. Furthermore, DEX exhibits a synergistic effect with opioids, potentiating their effect. The use of DEX with fentanyl requires careful monitoring of the patient’s hemodynamic status (15).

From a clinical perspective, our results indicate that both LIDO and DEX are effective ways to prevent FIC; nevertheless, the patient's hemodynamic status and clinical situation should be taken into consideration while making this decision. Patients who are hemodynamically unstable, hypovolemic, or at risk of hypotension, such as those who are elderly, have cardiovascular comorbidities, or are on antihypertensive drugs, may benefit more from LIDO. There is ample evidence of LIDO's safety and effectiveness in reducing FIC with negligible hemodynamic changes (4, 12, 16, 29). In contrast, DEX may be more suitable for patients who are hemodynamically stable and may benefit from its additional sedative and anxiolytic properties (10). However, as demonstrated in our study and supported by previous research, DEX can cause significant reductions in heart rate and blood pressure through suppression of norepinephrine release (9, 10, 28). Furthermore, in clinical scenarios where rapid drug administration is required, LIDO offers a practical advantage due to its shorter pre-administration time (90 seconds before fentanyl injection) compared to DEX (10 minutes before induction). Therefore, in emergencies or time-sensitive situations, LIDO appears to be the more practical choice (4, 12).

Clinicians should also consider that DEX exhibits a synergistic effect with opioids, which may potentiate their effects (10). Consequently, when DEX is selected, enhanced monitoring of heart rate, blood pressure, and oxygen saturation is recommended, especially during the induction phase. In summary, while both agents demonstrate comparable efficacy in preventing FIC, LIDO may serve as a safer first-line option in routine clinical practice due to its favorable hemodynamic profile, whereas DEX should be reserved for selected patients who can tolerate its cardiovascular effects and may benefit from its sedative properties.

There are a number of limitations to this study that should be noted. First, the study was carried out at a single facility, which can restrict the results' applicability to different demographics or clinical contexts. Second, although gender-related variations in airway sensitivity have been shown in earlier research, the majority of participants were men, and the low representation of women may have affected the overall incidence of FIC. Third, no long-term monitoring was done to assess delayed side effects or postoperative results. Lastly, this trial did not evaluate the dosage-response relationships for LIDO and DEX, which could offer more information about the best ways to dose drugs. To confirm these results, more multicenter research with a balanced gender distribution and dose-response assessment is advised. In addition, the small number of female patients may have potentially influenced the results. We suggest investigating the effects of DEX and LIDO at varying doses and forms in multicenter randomized controlled trials with balanced gender distribution.

In conclusion, both LIDO and DEX are effective in preventing FIC. However, hemodynamic parameters are more markedly suppressed in the DEX group.

## Data Availability

The dataset presented in the study is available on request from the corresponding author during submission or after publication.

## Clinical Trial Registration Code:

The study was registered at the Iranian Registry of Clinical Trials under the code IRCT20211023052848N1 on November 17, 2021.
